# Bioengineered bioreactors: a review on enhancing biomethane and biohydrogen production by CFD modeling

**DOI:** 10.1080/21655979.2021.1972195

**Published:** 2021-09-17

**Authors:** Anand Kumar Saini, Tanja Radu, Kunwar Paritosh, Vinod Kumar, Nidhi Pareek, Dharmendra Tripathi, Vivekanand Vivekanand

**Affiliations:** a Centre for Energy and Environment, Malaviya National Institute of Technology, Jaipur, Rajasthan, India; b School of Architecture, Building and Civil Engineering, Loughborough University, Loughborough, UK; c Bioenergy and Resource Management Centre, School of Water, Energy and Environment, Cranfield University, Cranfield, UK; d Department of Microbiology, School of Life Sciences, Central University Of Rajasthan, Bandarsindri, Kishangarh, Ajmer, Rajasthan, India; e Department of Mathematics, National Institute of Technology Uttarakhand, Srinagar, India

**Keywords:** CFD simulation, methane, bioenergy, biomass, biogas, power consumption

## Abstract

Computational fluid dynamics (CFD) is numerical strategy developed for simulating the behavior of liquid and gas flow. CFD may be applied starting from aerospace, engine design, vehicle aerodynamics, power plants and chemical industries for analyzing and solving relevant system design and process issues. Biogas produced during anaerobic digestion (AD) is sustainable and renewable alternative to fossil fuels. AD may improve the controlled production of biogas and offers significant environmental benefits. This review focuses on research outcomes relevant for enhanced biogas production by exploring the possible applications of CFD in AD technology. CFD-related research performed in AD conditions in order to improve mixing performance, reduce power consumption, and understand the effects of total solid (TS) concentrations on flow behavior have been discussed. In addition, the use of AD for bio-hydrogen production, wastewater treatment, and sludge treatment are looked in. This review also identifies novel areas for AD technology advancement where there is potential for economic improvement in renewable energy production. Finally, future research needs have been identified, focusing on the opportunities to integrate conceptual and mathematical models for advancing CFD simulations for bioenergy.

## 1. Introduction

Demand for energy continues to grow as the world population increases alongside expectations of improved living standards. The result is increasing energy demand including conventional sources, i.e., oil, coal, and natural gas. These fuels cannot be considered sustainable options as fossil reserves are finite. Using fossil fuels is increasingly damaging the environment by polluting emissions of CO_2_. Increasingly, electricity demand is being met by solar and wind energy, whereas meeting transportation and heating needs shows that there are more opportunities that may be fulfilled using bioenergy. Bioenergy includes all fuels produced by living organism, e.g. biogas, biodiesel, bioetahnol etc. and are thus renewable. Substantial research has been reported on biomass, pretreatment, biofuel production and parameters affecting the production of biofuels. Biogas is a mixture of carbon CH_4_ and CO_2_ in varied proportions (approximately in ratio 60:40) and typically includes other gases in low concentration. Once processed (CO_2_ scrubbing and removal of other impurities) it has potential to replace natural gas, as purified biomethane of at least 98% can be injected to gas grid. Where CH_4_ concentrations are high and trace compounds low, this also has potential for direct use in kitchens and natural gas-fueled vehicles after purification and bottling. Biogas can also be used to produce renewable electricity [1] thus presenting a viable option to address energy demands as a fossil fuel alternative.

To produce biogas, biomass material in liquid, slurry or solid form, plus water and inoculum where required are fed into a closed vessel to achieve anoxic conditions. The organic matter referred to as biomass decomposes in the absence of oxygen through a four-stage fermentation process of anaerobic digestion (AD) [2]. Degradation during AD is due to the diverse microbial population present in the digester [3]. A wide number of parameters affect the performance of AD including material characteristics of the feedstock, pH of the substrate [4], digester temperature, mixing, feeding pattern, hydraulic retention time [5] and the population and distribution of microbial communities inside the digester, e.g. bacteria and archaea.

Basic commercial requirements for digester operation include managing the organic loading rate (OLR), optimizing the reactor volume and hydraulic retention time (HRT), whilst maximizing methane yield [6]. To fulfil these requirements, a number of factors have to be considered such as reactor design and the extent to which mixing is necessary to enhance methane production. According to Van Hulle et al. [7], a ~10% reduction in methane yield was observed when mixing was stopped for a pilot scale digester. In contrast, a lab-scale digester showed no significant difference in methane yield for both mixed and unmixed conditions. Karim et al. [8,9] report that mixing enhances biogas production in digesters only when thick slurry is fed, i.e. 10% to 15% manure slurry, whereas for thin slurry mixing is not required. This shows that biomass viscosity, determined by its concentration, influences the need to mix which in turn influences the performance efficiency of the AD. Further parameters that influence the processing rate of AD and thus biogas yield include the agitation speed, loading rate, geometrical design, and temperature distribution. These parameters have to be chosen within specific limits based on theoretical calculation and experimental results [10,11]. Recently, a number of relevant parameters including the optimization of mixing speeds have been studied using simulation tools including computational fluid dynamics (CFD) along with experimental validation to improve the performance of digesters at pilot scale.

CFD is a popular and efficient tool for mathematically simulating the flow and behavior of fluids. It is widely used and accepted in many sectors. Applications for CFD analysis include heating ventilation and air conditioning (HVAC), the aerospace industry, vehicles aerodynamics, and building design. CFD in industries is used to predict flow and pressure change, as well as noise generation and changes in temperature [12]. The analytical scope in industry differs for each application. Examples for the HVAC industry include cooling/heating load analysis, evaluation of thermal comfort and duct design [13–17]. In aerospace, CFD is used to predict the flow of air past the aircraft body and wings, hence CFD is used for body and wing design [18]. CFD for vehicle aerodynamic in mass-production as well as high performance cars is already well established and undergoing further improvement. Similarly, applications to ensure efficient building design for thermal comfort, ventilation, and airflow are evident [19,20]. Amongst many other application areas, simulations for wind power [21,22], nuclear power [23], and coal-biomass co-firing [5] are reported.

Application for CFD simulations of AD have become popular as it allows the flow of feedstock substrate, the vortex motion of substrates from agitation, and gas production to be visualized. Simulation is also useful to determine the location of eddy formations. Operating conditions including power consumption, OLR, mixing rates, temperature distribution all form part of the digester design and optimization process. These factors can then be used to define the optimal digester retention time thus maximizing the economic value of biogas production.

Unlike many production processes, the nature of the working fluid of AD is a highly variable mixture of solid, liquid and gas phases. As the working fluid changes, the simulation strategy must recognize this variation and its effect on the overall process. Thus, data inputs that reflect the process include the suspension of solid particles, the movement of gas bubbles and power consumption. This review focuses on AD-applied CFD research relevant to the operating parameters in this context.

## 2. Computational fluid dynamics software

Common commercially available for CFD modeling packages include ANSYS-Fluent, ANSYS-CFX, PHOENICS, CFD2000, and Star-CFD. Governing equations including the conservation of mass, conservation of momentum, and conservation of energy define the foundations within CFD. These are then used to solve equations that predict flow behavior, temperature distribution, the pressure profile, and species concentrations [24].

**Figure 1.**
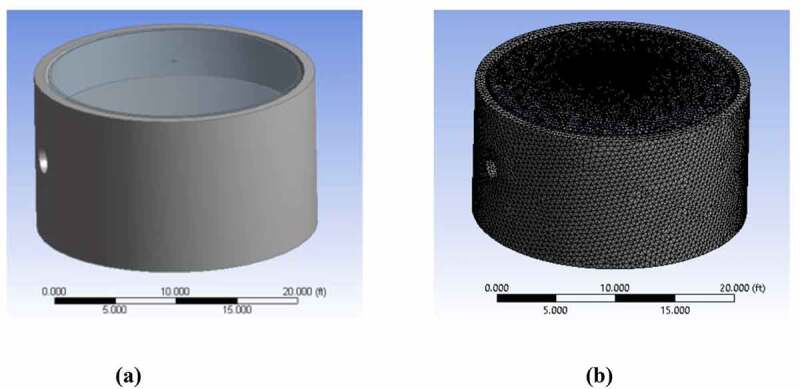
a) Geometrical model, and b) generated mesh of a floating dome digester (developed in ANSYS software)

In simulating fluid flow; the first step from defining the optimization goal is in listing the assumptions to be taken into account to simplify the simulation. A geometrical model of the system is then prepared within which to analyze the flow behavior. From this preparation of model geometry, mesh generation is used to sub-divide the model into n-number of pockets. Mesh generation is necessary to set cell zone and boundary conditions so that a solver can run which predicts the flow conditions between each point within the system. Figure 1 shows the geometrical and mesh model of a representative floating dome type anerobic digester. Software for geometry and mesh design includes Design Modular (DM), CAD, Meshing mode fluent, and ANSYS SpaceClaim geometry.

Followings are the model preparation and mesh generation solver settings and physical models then need to be defined. In this step, selection of a numerical solver based on pressure and density, the definition of materials, type of flow, i.e., laminar or turbulent, and the turbulence mode are defined. Boundary conditions that specify different wall conditions for the digester and agitator are also explained, and simulation is then performed, which allows setting up controls for the solver to initialize the fluid flow.

**Figure 2.**
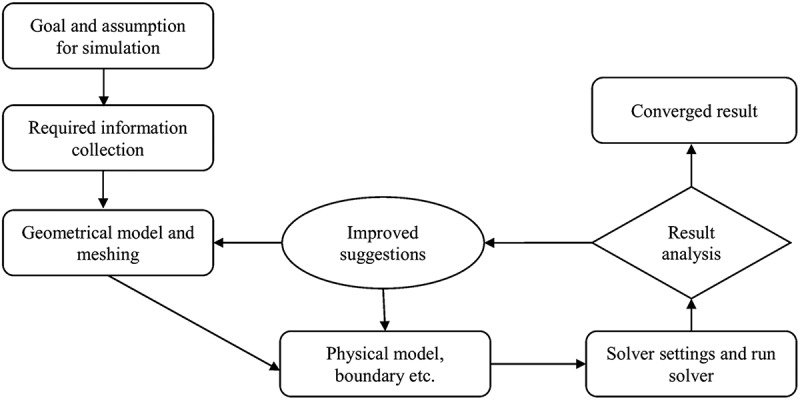
Flow-chart of CFD strategy using ANSYS Fluent

Once the simulation is completed, the software provides solutions in different forms via velocity contours, temperature, turbulent kinetic energy, etc. Iterations are also required to reach a convergent solution. This step is commonly called post-processing. A typical flow-chart of CFD strategy using ANSYS Fluent software is depicted in Figure 2.

### 2.1. Assumptions for simulation

A series of assumptions are taken into consideration for software simulations. These are important for the sake of simplicity and defined before starting. Common assumptions for bioenergy simulation are given below [25–29]:

- Liquid phase such as slurry, i.e. mixture of substrate, sludge and water etc. is homogeneous and incompressible.
- Components of the liquid phase share the same mean velocity, pressure and temperature fields.
- Biogas bubbles are uniformly distributed in the liquid phase.
- Fluid properties such as density and dynamic viscosity are specified according to measurement data, and then fixed.
- Type of fluid: Newtonian or non-Newtonian.
- Flow is turbulent.
- Constant temperature inside the digester, if heat transfer effect is neglected.

### 2.2. Governing equations required for CFD simulations

#### 2.2.1. Continuity equation

The equation based on the principle of conservation of mass is called the continuity equation. For the continuous flow of any fluid, it must satisfy the continuity equation.

The equation for three-dimensional unsteady state fluid flow [2]:

(1) $$\nabla .\left(\vec{v}\right)+\frac{\partial }{\partial t}\left(\rho \right)=0$$

For three-dimensional incompressible fluid flow:

(2) $$\frac{\partial }{\partial x}\left(u\right)+\frac{\partial }{\partial y}\left(v\right)+\frac{\partial }{\partial z}\left(w\right)=0$$

For one dimensional flow: $\rho_{1}A_{1}V_{1}=\rho_{2}A_{2}V$ (1 and 2 represent two positions in flow)

Where, ∇ is $\frac{\partial }{\partial x}+\frac{\partial }{\partial y}+\frac{\partial }{\partial z}$, $\vec{v}$ is three-dimensional velocity vector written as $ui+vj+w\dot{k}$, *ρ* is density.

#### 2.2.2. Conservation of momentum

The following equation defines the conservation of momentum for an inertial reference frame [3,25,30]:

(3) $$\frac{\partial }{\partial t}\left(\rho .\;\vec{v}\right)+\nabla .\left(\vec{v}.\vec{v}\right)=-\nabla p+\nabla .\left(\tau \right)+\vec{\rho g}+\vec{F}$$

Here τ is stress tensor given by

(4) $$\overset{=}{\tau }z\left[\left(\nabla \vec{v}+\nabla {\vec{v}}^{T}\right)\right]-\frac{2}{3}\nabla .\vec{v}I$$

*p* is static pressure, $\vec{\rho g}$ is gravitational body force and $\vec{F}$ is external body force, z is viscosity, *I* is the unit tensor.

#### 2.2.3. Conservation of energy

Conservation of energy is described by [3,31]:

(5) $$\frac{\partial }{\partial t}\left(\rho E\right)+\nabla .\left\{\vec{v}\left(\rho E+p\right)\right\}=-\nabla .\left(\underset{j}{\sum }h_{j}J_{j}\right)+S_{h}$$

*E* is for energy (KJ), $h_{j}$ is enthalpy, $J_{j}$ is diffusion flux, and *S_h_* is user defined function.

#### 2.2.4. Turbulence model

Different turbulence models are available in CFD to simulate the mechanical mixing of a fluid inside a digester such as standard k-ε model, RNG k-ε model, realizable k-ϵ model, standard k- ω model, SST k-ω model, and Reynolds stress model. Standard k-ε model is usually used for studies due to its simplicity.

The standard k-ε model gives a good extrapolation for mean velocity and pressure [32]. This model is based on transport equations for turbulence, kinetic energy (k) and its dissipation rate (ε). The standard k-ε model is valid only for fully turbulent flows where molecular viscosity effect is neglected. The transport equation for the standard k-ε model can be given by the following equation for turbulence where kinetic energy k, and its rate of dissipation ε are obtained by using the transport equation as below [28,30,33]:

(6) $$\frac{\partial }{\partial t}\left(\rho K\right)+\frac{\partial }{\partial x_{i}}\left(\rho ku_{i}\right)=\frac{\partial }{\partial x_{j}}\left[\left(\mu +\frac{\mu_{t}}{\sigma_{k}}\right)\frac{\partial k}{\partial x_{j}}\right]+G_{k}+G_{b}-\rho \varepsilon -Y_{m}+S_{k}$$

and,

(7) $$\frac{\partial }{\partial t}\left(\rho \varepsilon \right)+\frac{\partial }{\partial x_{i}}\left(\rho \varepsilon u_{i}\right)=\frac{\partial }{\partial x_{j}}\left[\left(\mu +\frac{\mu_{t}}{\sigma_{\varepsilon }}\right)\frac{\partial \varepsilon }{\partial x_{J}}\right]+C_{1\varepsilon }\frac{\varepsilon }{k}\left(G_{k}+C_{3\varepsilon }G_{b}\right)-C_{2\varepsilon }\rho \frac{\varepsilon^{2}}{k}+S_{\varepsilon }$$

Where, $G_{k}$ denotes the generation of turbulence kinetic energy due to the mean velocity gradients, $G_{b}$ is the generation of turbulence kinetic energy due to buoyancy, *Y_m_* is the contribution of the fluctuating dilatation in compressible turbulence to the overall dissipation rate. $C_{1\varepsilon },C_{2\varepsilon }$, and $C_{3\varepsilon }$ are constants, $\sigma_{k}$ and $\sigma_{\varepsilon }$ are the turbulent Prandtl numbers for *k* and ε, respectively. $S_{k}$ and $S_{\varepsilon }$ are user-defined source terms.

### 2.3. Selection of turbulence model

Flow inside the digester is generally considered as turbulent flow. In CFD, the flow is defined by selecting an appropriate model for turbulence. Selection of an appropriate model depends on the component of primary interest. Wu [34] studied six different turbulent models, i.e. standard k-ε model, RNG k-ε model, realizable k-ϵ model, standard k- ω model, SST k-ω shear stress transport model, and Reynolds stress model, for the mechanical mixing of non-Newtonian fluids. Of these six models, the realizable k-ε model and the standard k-ω models were the most appropriate to describe the mechanical agitation of a non-Newtonian fluid. Zhang et al. [35] found the standard k-ε model as the more appropriate turbulence model based on power number. For the standard k-ε model the error indicator from its power number was the minimum. Wu [30] found the SST k-ω model economical for two-phase flow as the prime concern for the study was to find where flow takes place in the core region, i.e. away from walls.

## 3. Parameters of biomass slurry affecting flow behavior

Basic fluid parameters such as density, kinematic viscosity, specific heat, TS concentration, and particle size distribution, have to be defined to simulate any model using CFD [36]. The further parameters of fluid type are also required. These parameters are discussed below in the context of defining a slurry or raw material feedstock in an anaerobic digester.

### 3.1. Rheology

Fluids can be broadly classified into two types 1) Newtonian and 2) non-Newtonian fluids. Newtonian fluids are those that obey Newton’s law of viscosity (e.g., water, oil, petrol, diesel, etc.). These are fluids where the viscosity does not change with time and deformation. The following equation gives Newton’s law of viscosity [2]:

(8) $$s=z.\frac{dv}{dy}$$

*s* is shear stress, *z* is viscosity, *v* is the velocity and *y* is the distance of layer from the reference plane in the y- direction.

Non-Newtonian fluids are those whose viscosity changes with time or deformation and thus do not follow Newton’s law of viscosity. Non-Newtonian fluids follow the Power law [28,37], given below:

(9) $$s=A.{\left(\frac{dv}{dy}\right)}^{n}$$

(10) $$s=z_{app}.\left(\frac{dv}{dy}\right)$$

Here,

(11) $$z_{app}=A.{\left(\frac{dv}{dy}\right)}^{n-1}$$

The viscosity of a non-Newtonian fluid is given by z_app_ (apparent viscosity). This varies with deformation in the fluid. The apparent viscosity of the non-Newtonian fluid is highly dependent on the total solids (TS) concentration of the feedstock [36]. Non-Newtonian fluids are further classified as Dilatant, Pseudo-plastic and Bingham-plastic.

**Figure 3.**
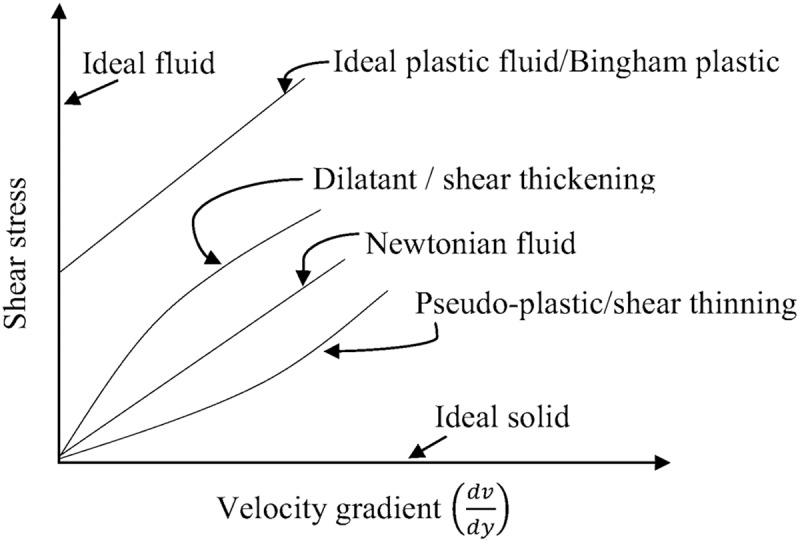
Different types of fluid and viscosity patterns

Dilatant or shear thickening fluids are those whose viscosity increases as deformation in the fluid increases such as honey. Pseudo-plastic or shear-thinning fluids are those whose viscosity decreases as deformation of the fluid is increased such as milk, blood, sewage sludge [6], animal manure [36] etc. Digested sludge is also a shear-thinning fluid. However, this behaves like a viscoelastic fluid when the shear stress is low. Sludge also shows instability in flow when the shear stress is low, i.e. appearing as shear bending [4]. Bingham-plastic fluid is a fluid that requires a force to start the flow such as a tooth-paste. Figure 3 shows the variation in viscosity against the velocity gradient for different fluids.

Slurry used in digesters to produce biogas is also a fluid; it may be Newtonian or non-Newtonian. A few authors assume biomass slurry to be a Newtonian fluid [31], others assume it is non-Newtonian [28,30,34]

The density of manure is largely dependent on its TS concentration. Landry et al. [36,38] reported that density increases as the TS percentage increases in manure. Similar values for density were also observed by Wu and Chen [39]. Rheological properties and densities of liquid cattle manure are shown in Table 1.

**Table 1.** Rheological properties and densities of liquid cattle manure at 35° C [1,39]

TS %	K (Pa s^n^)	n	γ (s^−1^)	z_min_ (Pa.s)	z_max_ (Pa.s)	ρ (Kg/m^3^)
2.5	0.042	0.710	226–702	0.006	0.008	1000.36
5.4	0.192	0.562	50–702	0.01	0.03	1000.78
7.5	0.525	0.553	11–399	0.03	0.17	1001.00
9.1	1.052	0.467	11–156	0.07	0.29	1001.31
12.1	5.885	0.367	3–149	0.25	2.93	1001.73

TS% = total solid percentage, K = consistency coefficient (Pa s^n^), n = power law index, γ = rate of shearing (s^−1^), z_min_ and, z_max =_ minimum and maximum viscosity (Pa.s), ρ = density (Kg/m^3^)

## 4. CFD application in the bioenergy sector

Since CFD is a useful tool to predict the flow behavior of fluid flowing into a system its application is increasingly popular in the bioenergy field. A number of flow types take place from feeding raw substrate to the flow from biogas production to the flow of slurry. By using CFD, mixing performance may be improved. Analyzing the flow of slurry within pipelines and digesters enables the design of the process to be improved, thus achieving the higher gas yields. Power consumption may also be reduced using CFD simulation. CFD simulation can also be used in wastewater treatment, sludge treatment AD and bio-hydrogen production to predict the effect of TS on velocity magnitude, mixing parameters and settling rate of solid particles. Therefore, these simulation studies relevant to the bioenergy sector are discussed.

### 4.1. Improving mixing performance

Few research reports focus on improving the mixing performance of biogas digesters using CFD simulation [25,28,30,40–42]. Biogas production is enhanced when thorough mixing is achieved. Effective biomass digestion depends on a number of factors such as: ensuring close contact between activated micro-organisms with freshly biomass feedstock, keeping solid particles in suspension, and increasing the mass transfer of by-products [43,44] and maintaining a constant temperature throughout the digester [30]. Importantly, mixing or agitation also drives out gas bubbles, avoids sedimentation of denser particulate matter and prevents the formation of floating and settling layers [45]. Mixing does not have been continuous and can be scheduled intermittently several times a day or hour [46].

A number of mixing methods are available, these include; biogas recirculation, mechanical impeller mixing and slurry recirculation [8]. Most widely, mechanical agitators driven by an electric motor are used. About 7–8% of total electric energy produced by biogas plant is consumed within the process. Of this energy demand, approximately 40% is used in digester mixing [47]. Energy consumption depends on the agitation speed, type and number of blades on the impeller, plus the TS concentration. Hence; understanding the influence of these variables can ensure that energy consumption is balanced effectively against the energy gain from biogas production where enhanced by agitation. Energy consumption to ensure mixing is important as low agitation speeds can result reduced effectiveness of the digester. Similarly, higher speed can result in high shear stresses causing internal damage to microbial cells and sludge flocs [25,48,49] resulting in a potential decrease in biogas production.

#### 4.1.1. Mechanical mixing

Mechanical agitation is commonly used for simplicity. An impeller with a fixed number of blades is connected to an electrical motor that rotates inside the digester. The number of blades on the impeller, its location, particle size, solid loading and agitation speed are factors that have the potential for contributing to increase the biogas production whilst reducing overall energy consumption [40].

#### 4.1.2. Agitator

**Figure 4.**
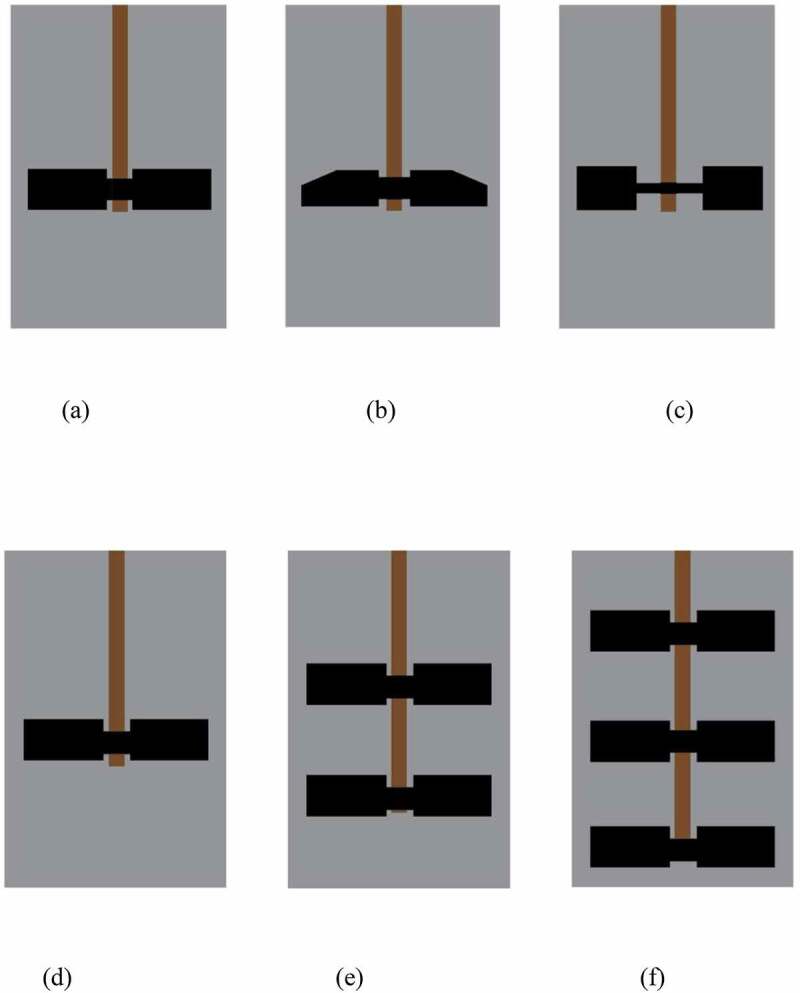
Types of blades. a) Pitched blade, b) High-efficiency blade, c) Disc-mounted flat blade, and Types of Impeller. d) Single impeller, e) Double impeller, f) Triple impeller

Shen et al. [28] investigated three types of pitched blade impeller including a high-efficiency blade and disc-mounted flat blade and reported on the efficiency of the triple impeller with a pitched blade. Figure 4. shows different types of blade and impeller, ibid [28]. Other types of impellers include the double four-blade Rushton impeller [25] and a helical impeller [50]. Yu et al. [51] suggested the helical ribbon impeller for high viscosity or high TS concentration and the A-310 impeller for low TS concentrations. Table 2 shows different studies reported in literature related to optimizing mechanical mixing strategy using CFD simulation tool.

**Table 2.** Different studies related to optimizing mechanical mixing strategy using CFD simulation at Lab scale

Digester	Slurry/raw material	Impeller	Speed range	Optimized speed	Output	Reference
Lab scale horizontal CSTR	Normal molasses, (51% sugar), water	Double four blade Rushton impeller	20, 30, 40, 50, 60, 70 rpm	50 rpm	Bio-hydrogen	[25]
Lab scale CSTR	Rice straw	Triple impellers with pitched blade	20, 40, 60, 80, 100, 120, 140, 160 rpm	80 rpm	Biogas	[28]
High solid anaerobic digester (HSAD)		A-310 impeller and helical ribbon				[51]
Lab scale CSTR	Normal molasses (53% sugar), water activated sludge	Two blade impeller having blade angle 45°	50, 70, 90, 110, 130 rpm	50–70 rpm	Bio-hydrogen	[65]
Egg shape anaerobic digester		A propeller having dual helical blades	400 rpm to 750 rpm	600 rpm	Biogas	[42]

#### 4.1.3. Agitation speed

Shen et al. [28] used CFD reports on suitable agitation parameters for efficient biogas production using rice straw as the raw material for AD and reported speeds of ~80 rpm as optimal for good mixing and achieving a high level of biogas production. Researchers then validated the CFD model with experiments using the same design.

Ri et al. [25] performed experiments with CFD simulation for a horizontal continuous stirred tank reactor (HCSTR) for bio-hydrogen production. It was observed that 50 rpm was the optimal agitation speed matched with enhanced bio-hydrogen production.

#### 4.1.4. Particle size

Mixing performance depends on the particle size of biomass, the extent to which biomass is in liquid form and the extent of solid particles in suspension that is then available for biogas production within the digester. For better microbial contact of particles, it is necessary to maintain the movement of the particles in suspension. Murthy et al. [40] reported that as size of particles increases, so does the settling velocity. As a result, homogeneity in suspension decreases. Therefore, high mixing speed is required to maintain the suspension of particles in the digester. Smaller the particle size, larger the surface area and thus higher the biogas production would be from the feedstock.

### 4.2. Reduction in power consumption

Typical power consumption for digester agitation varies from 10 to 100 Wh.m^−3^ [46], depending on the process type, agitator design, TS concentration and composition of the feed-stock [52]. Power consumption can be represented using a power number, i.e. a dimensionless number that characterizes the agitator [35]. Power numbers can be calculated in CFD simulation by using a turbulence model. The rheological properties of the digester slurry are influential in this calculation. Yu et al. [51] observed and reported the increase in the power number with an increasing percentage of TS concentration. Notably, the number increases with decreasing Reynolds number (Re) [51]. The power number in CFD is given by the following equation [35,50].

(12) $$N_{p}=\frac{P}{\rho .N^{3}.d^{5}}$$

where,

(13) P=n.2πNT

N_p_ is the power number in CFD, P is the power input of agitator (W), n is the number of blades on the agitator, N is agitator speed (rps), and T is torque (N.m). The power number is also influenced by the fluid density. If *i* is the surface element, *Δp* is the pressure difference between the front and back side of the blade, *a* is the area (m^2^), and *r* is the radial distance of the element from the axis of rotation then the torque *T* may be calculated by:

(14) $$T=\underset{i}{\sum }{\left(p\right)}_{i}a_{i}r_{i}$$

Zhang et al. [35] used CFD simulation to examine the power consumption for the anaerobic mono- and co-digestion of cattle manure and corn stover. The group also explored two types of mixing mode, i.e., continuous and intermittent mixing. From simulation results it was observed that for different mixing modes the optimal feed ratio in co-digestion processes changes with net power production. Using a CFD simulation validated with experimental data Taghavi et al. [53] predicted the power consumption for a dual impeller, a six blade Ruston turbine in a stirred tank. Power consumption for the bottom impeller was higher than upper impeller.

### 4.3. Effect of TS on flow behavior

AD slurry contains substrate and water. Properties like density, viscosity depend on TS concentration of the slurry. Higher TS concentrations in the slurry results in higher levels of cohesion amongst the molecules, thus in turn resulting in increased viscosity. To balance this cohesion force, a higher force is required. This results in decreased mixing intensity [34]. Increased mixing intensity increases homogeneity within the digester. Hence, as TS percentage increases the digester mixing speed to maintain homogeneity [54]. The presence of higher cohesion and need for high mixing intensity result in increased power consumption [55]. TS concentration in the AD also influences the temperature uniformity, pH and effectiveness of micro-organism in the decomposition process [51].

The slurry used for AD is usually considered as a non-Newtonian fluid. Wu and Chen [39] performed experiments and simulation to understand the difference in flow behavior of Newtonian and non-Newtonian fluids. Results described the flow path of Newtonian fluid as being along the right-sidewall toward the topside, and for non-Newtonian fluid, it was along left-sidewall approaches toward the topside. As the magnitude of velocity increases, the flow pattern remains same. A similar kind of study was performed by Wu [30] using gas recirculation as the mixing technique also observed a decreased efficiency. Meroney and Colorado [56] simulated circular anerobic digester of different diameter (13.7, 21.3, 30.5, and 33.5 m) with mechanical mixing using draft tube impeller agitator and reported the maximum HRT for a digester of diameter 33.5 m, with an active volume for impeller diameter 30.5 m.

## 5. Miscellaneous use of CFD in AD

CFD simulation can widely be used in predicting the mechanical mixing of the AD to see the flow pattern of fluid, velocity magnitude and gas substrate separation etc. Extensive studies have been performed for simulating AD in addition to the above-mentioned fields, for processes such as plug-flow digesters, up-flow anaerobic sludge beds (UASBs), and expanded granular sludge beds (EGSBs). Software, analytical approaches, turbulence and fluid models used for CFD studies to analyze and improve mixing strategies and process parameters for these sectors are summarized in Table 3.

**Table 3.** Different software, approaches, turbulence model and fluid used for CFD studies to improve various mixing strategies and other relevant parameters

CFD Software	Physical model and approach	Turbulence model	Type of mixing	Type of fluid	Reference
Fluent 14.5	Eulerian, multiple reference frame (MRF)	Standard k-ε	Mechanical	-	[25]
Fluent 16.2		Realizable k-ϵ and standard k-ω	Mechanical and pumped recirculation	Newtonian and non-Newtonian	[50]
Fluent 6.3	MRF approach		Mechanical	Non-Newtonian	[28]
Fluent 12.0	MRF approach	standard k-ω and the realizable k-ϵ models	Mechanical	Non-Newtonian	[34]
Fluent 12.0	Eularian multiphase flow model	SST K-ω	Gas recirculation	Non-Newtonian	[30]
Fluent 6.3	MRF approach	Realizable k-ϵ models	Mechanical draft tube	Non-Newtonian	[42]
Fluent 6.3		Standard k-ε	Mechanical		[56]
Fluent 6.2	Eulerian multi-fluid model, MRF	Standard k – ε	Mechanical		[40]

### 5.1. Recirculation mixing

Meister et al. [50] performed investigations into mixing Newtonian and non-Newtonian fluids in an AD using pumped recirculation and impeller mixing. An issue was observed with pumped recirculation that it mixes fluid in the plane parallel to the feeding pipe efficiently but does not mix fluid in the plane perpendicular to feed input pipe properly.

Vesvikar and Al-Dahhan [41] performed three-dimensional CFD simulations to mimic the AD. A sparging gas stream at different flow rates was used for mixing within the digester. Simulation for different configuration of the digester was also performed. It was observed that the conical bottom digester design helps to increase the effective working volume of the digester and thus enhances biogas production, unlike the flat bottom unit. Wu [30] also performed the simulation for draft tube gas recirculation mixing with different TS concentration and reported that as the TS concentration increases the intensity of mixing decreases. So, for high TS concentration, it is recommended to use confined gas mixing instead of unconfined for the uniformity.

Wu [42] developed a CFD model to characterize an egg-shaped anerobic digester. Different concentrations of fluid were evaluated by using a propeller for mechanical mixing of the fluid. Comparison of different strategies of mixing was also taken into the account. It was reported that mechanical mixing is better than external pumped recirculation. Egg shape of digester provides higher efficiency as compared to cylindrical shape digester, which may be due to the fact that the travel time of particle before settling is increased as the bottom of egg shape digestor is curved.

### 5.2. Plug flow digester

Plug flow digester is the combination of a horizontal fermenter and a continuous stirred tank reactor (CSTR). tt is often used for AD of feedstocks with high viscosity. Most of the agricultural lignocellulosic biomass has high solid content and thus possess high viscosity. Hence, the plug flow digester is commonly used for AD of these kinds of feedstocks. Lübken et al. [57] performed the CFD simulation for a plug flow digester to observe the axial mixing and evaluate different arrangements of agitators to see the resultant flow pattern. The reported model also showed that blending machine with the arrangement of the vertical paddles at of 120 degrees may lead to develop strong horizontal component in the flowing fluid. However, the changes could be made in the patterns and arraengements in the device so that there is reduction in it.

Wu et al. [31] developed three-dimensional CFD simulation for a plug flow AD to predict the biogas production and compared with one biogas production data measured by Gebremedhin et al. [58]. The CFD model study was based on mass, energy conservation, and species transfer. No turbulence model was used as velocity kept was very low.

### 5.3. AD in sludge treatment

CFD simulation has also been used in AD of sewage sludge to evaluate the mixing performance and flow behavior inside the digester. Bridgeman [6] simulated a laboratory scale AD for mixing of sewage sludge and developed flow field of sewage sludge by taking five different total dissolved solid (TDS) content sludge (2.5, 5.4, 7.5, 9.1, and 12.1%) at the mixing speed of 100 rpm. The results showed that the velocity was decreasing as TDS percentage of sludge increased for same power consumption. Craig et al. [59] also developed a CFD model for AD of sewage sludge. An impeller located in a draft tube was used for mechanical mixing and investigating the effect of the rheology of sewage sludge on the performance of digester. It was found that rheological behavior of sludge affects both the torque required for the impeller and mixing performance of digester.

A study on AD of highly degradable organic content of solid waste was done by Yu et al. [60]. A model of high solid AD system was designed, and the model was able to foresee pH, volatile fatty acid and biogas production.

### 5.4. AD in bio-hydrogen production

Bio-hydrogen may be produced by AD of organic substrates such as a mixture of biomass (normal molasses) with activated sludge, wastewater, and sewage sludge. There are many chemical, biological and physical parameters that affect the efficiency of bio-hydrogen production. Biological and chemical factors are fermentation type, type of substrate used, pH of substrate, etc. Physical parameters include digester design, velocity field, shear stress distribution and turbulence intensity. These parameters affect the efficiency of production as being directly affecting the microbial community, settling rate of activated sludge, biomass activity etc. Optimizing any of above parameters may enhance bio-hydrogen production. Alkaline pre-treatment was used to enhance bio-hydrogen production by keeping the pH levels under observation [61–63]. Optimization of physical parameters may be done by the hydrodynamic study of the digester, necessary for increased levels of hydrogen production. CFD simulation may also be used to understand the hydrodynamics and chemical reactions hence improving the rate of production.

Wang et al. [64] done simulation of expanded granular bed sludge (EGSB) reactor for hydrogen production to see the hydrodynamics of EGSB for various hydraulic retention time (HRT). The group also reported the Hydrogen production in the range of 0–4 mol/mol-sucrose in expanded granular sludge bed (EGSB) reactor after CFD simulation. For 2D CFD simulation, Eulerian-Eulerian three-phase model was used. Results exposed that with long HRT solid–liquid-gas phase inside reactor shows very heterogeneous flow pattern. Hence, suitable HRT is necessary for high and economical hydrogen production.

Ding et al. [65] studied optimization of the impeller and its agitation speed. For this, the simulation of two-phase lab scale CSTR using three-dimensional CFD with experimental validation was performed. The results showed type and speed of impeller greatly influence bio-hydrogen production. By selecting optimal impeller and speed, hydrogen yield was maximized and startup time was minimized compared to the normal impeller. Wang et al. [66] done CFD simulation to compare lab scale CSTR with industrial scale CSTR for bio-hydrogen production and described some parameters to be optimized like velocity field and stagnation zone in industrial scale CSTR.

### 5.5. AD in wastewater treatment

In AD wastewater treatment, biogas and digestate are generated as a product. UASB, EGSB reactors are used to treat wastewater. UASB reactor is visualized as a number of CSTRs are set up in series. EGSB reactor is a UASB reactor with higher up-flow velocity and greater the height to diameter ratio, offers partial expansion to granular sludge bed. In EGSB, reactor effluent is recycled which further dilutes the influent [67].

Ren et al. [68] investigated hydrodynamic characteristics of UASB reactor. A Eulerian-Eulerian three-phase fluid approach was used to see the flow behavior in UASB reactor using a three dimensional CFD simulation. Pan et al. [67,69] investigated the hydrodynamics of EGSB reactor. A two-dimensional CFD with Eulerian three-phase fluid approach was used to see the effect of baffle angle on separation efficiency and hydrodynamic characteristic in the three-phase separation zone. Baffle angle of 40° was found to be the most efficient regarding best separation, and sludge loss rate was also smallest at this angle.

## 6. CFD simulation in bioenergy and its importance

Research work has been performed related to CFD simulation in bioenergy sector still, there are huge opportunities or areas where CFD study is sparsely reported and/or not performed so far. Below are the possible areas, where CFD simulation can be performed and incorporated in the system in near future to make the system more sustainable.

### 6.1. Heat transfer effect

Most of CFD simulation related studies in bioenergy sector are done so far are mainly based on mixing performance and power consumption [35,50,51]. By taking temperature as variable and providing atmospheric condition, heat transfer effect may also be predicted in CFD simulation tool. Using CFD simulation temperature distribution inside and across the walls of the digester, CSTR, etc. may be studied.

Wu and Bibeau [29] used CFD simulation for the underground digester to reduce the heating requirement for colder climate application. A 3D simulation model in CFD was developed and the model was used to optimize various geometrical parameters in order to reduce heat transfer from the bottom, walls, and top of the digester. The comparison of heat transfers among single, double, and quadruple tank configuration for the same working volume was reported in the article. Results showed that the heat transfer is increased by 11.5% for double tank reactor and 16.5% for four-tank reactor configuration as compared to the single tank configuration.

The temperature distribution also becomes important if the plant is located above the ground level and atmospheric temperature varies with seasons like Indian climate. India has current installed capacity of 383.3 Giga Watt (as on 31 May 2021), which is about to increase upto three times as of now.

Biogas production varies with temperature conditions inside the digester. Therefore, CFD becomes an important tool to see the temperature distribution inside disaster to enhance biogas production. Wu and Chen [55] performed CFD simulation for anerobic lagoons for a whole year to see the effect of temperature on methane production and reduction in biological oxygen demand (BOD). The reported results suggested that an insignificant effect on methane production was seen and the time taken in 99% BOD reduction is much more in January month as compared to July. This showed the effect of seasons (temperature) on AD process into the digester.

### 6.2. Mixing of substrate

Optimization of mechanical mixing by CFD simulation is the area where lots of work have been done [6,25,28,34,70]. However, there are more mixing strategies which are used for mixing of the substrate in digester like pumped recirculation, gas recirculation etc. are sparsely reported in the literature.

Mixing quality, mixing performance and power consumption etc. are the strategies may also be realized using CFD simulation tool. Flow comparison of different mixing strategies may also be done using simulation. Wu [30] did a comparison of these three mixing strategies and reported mechanical mixing is most efficient than gas recirculation and pumped recirculation is the least efficient among all the strategies.

### 6.3. Simulation of plant scale and real biogas digester

AD can be fruitful technology to treat organic waste and subsequently reduce the energy load on fossil fuels [71,72]. Simulations done so far in bioenergy sector are related to lab scale digesters (CSTR, UASB reactor, etc.). Similar approach of simulation can be used for an industrial, full-scale digester. By using simulation, the problems associated with the large-scale digester can be predicted and would be resolved either before commissioning the plant or during operation. Problems which may occur in a digester, are settling of suspended particles, uneven temperature distribution, and power loss. So, the CFD can be performed for plant scale digester or large anaerobic digester for biogas production to see the flow behavior, mixing, power consumption, heat transfer etc. By using CFD simulation input parameters for the digester, the speed of mixing, temperature conditions etc can be optimized. Other than these performance parameters design of digester, location of inlet-outlets, design of impeller etc. of a real digester are also can be predicted using CFD simulation. Biogas may be upgraded to biomethane after removal of carbon dioxide, H_2_S, water vapors and other impuritis by different methods like chemical scrubbing, water scrubbing, adsorption etc. Currently, in India these are commonly used and normal CSTRs are used for biogas production or various types of pre-fabricated biogas plants (fixed dome, floating dome etc) are available commercially for the same.

The increasing demand of energy worldwide and push for generation of maximum energy from renewable energy resources has led to very interesting findings. The fast development of computing technologies has come up with major role of CFD in AD for improved biogas production which is a renewable energy source. By the use of CFD, the visualization pattern of fluid flow, its relevant research and development has led to the basis for high yield of biogas as most of the limitations could be covered and/or could be solved with the help of CFD. This opens the new doors for the opportunity to go for deeper research in the flow behavior of biogas slurry inside the bioreactor and generate maximum amount of biogas which earlier was not. This will pave the way for future research on simulating the pattern, solving complex problems, and design new methods for harnessing maximum energy from AD process. CFD has great potential for improving the AD in terms of study on mixing time, viscosity, dead zones, TS concentration, reduce power consumption, continuous or intermittent mixing technology etc in near future which may further be explored.

## 7. Conclusions

This review summarizes types of research done on CFD simulation of AD for biogas and bio-hydrogen production, AD of sewage sludge, and CFD application in bioenergy. CFD simulation is the useful tool to evaluate the hydrodynamics related to bioenergy systems. CFD is also helpful in optimizing certain parameters such as agitation parameters, design parameters of digester etc. to minimize the power consumption and to improve the efficiency of the digester. Mixing inside the digester improves performance of digester to maximize the working volume of the digester, and to trap out the gas produced and enhancing biogas production. Therefore, optimization of mixing parameters is necessary to minimize power consumption because a large amount of energy, about 40% of total produced electric energy, is consumed in agitation sector. Generally, for simulation of bioenergy systems, fluid is considered as non-Newtonian fluid, and Eulerian-Eulerian multiple reference frame approach with a turbulence model is selected. By analyzing the results of the simulation, optimum agitation speed, stagnation zone, TS concentration and the viscosity of the fluid can be chosen to enhance the performance of anaerobic digester. The simulation may also help to see the heat transfer, mass transfer from the digester. There are few more sectors related to bioenergy such as gasifiers, bio-refinery, biodiesel etc. in which simulation were used. Further, research opportunities will also be available related to CFD simulation in these bioenergy sector to predict the flow pattern, temperature and optimization of different processes of gas generation (e.g. methanogenesis), in order to enhance biogas or bio energy production.
